# A Secure LEACH-PRO Protocol Based on Blockchain

**DOI:** 10.3390/s22218431

**Published:** 2022-11-02

**Authors:** Ghada Sultan Aljumaie, Wajdi Alhakami

**Affiliations:** Department of Information Technology, College of Computers and Information Technology, Taif University, P.O. Box 11099, Taif 21944, Saudi Arabia

**Keywords:** security, wireless sensor networks, LEACH, blockchain, ECC, BAN logic, AVISPA

## Abstract

Wireless Sensor Networks (WSNs) are becoming more popular for many applications due to their convenient services. However, sensor nodes may suffer from significant security flaws, leading researchers to propose authentication schemes to protect WSNs. Although these authentication protocols significantly fulfill the required protection, security enhancement with less energy consumption is essential to preserve the availability of resources and secure better performance. In 2020, Youssef et al. suggested a scheme called Enhanced Probabilistic Cluster Head Selection (LEACH-PRO) to extend the sensors’ lifetime in WSNs. This paper introduces a new variant of the LEACH-PRO protocol by adopting the blockchain security technique to protect WSNs. The proposed protocol (SLEACH-PRO) performs a decentralized authentication mechanism by applying a blockchain to multiple base stations to avoid system and performance degradation in the event of a station failure. The security analysis of the SLEACH-PRO is performed using Burrows–Abadi–Needham (BAN) logic and Automated Validation of Internet Security Protocols and Applications (AVISPA) tool. Moreover, the SLEACH-PRO is evaluated and compared to related protocols in terms of computational cost and security level based on its resistance against several attacks. The comparison results showed that the SLEACH-PRO protocol is more secure and requires less computational cost compared to other related protocols.

## 1. Introduction

Wireless Sensor Networks (WSNs) have gained the attention of many researchers due to the rapid development of wireless technology and embedded systems. WSNs have less expensive sensors, small sizes, and low processing and computational requirements. Nodes that use sensors collect and measure data from the network environment. Wireless sensor (WS) nodes consist of radio transceivers, embedded processors, storage devices, power sources, and sensors [1,2]. WSNs have a variety of applications, such as tracking and monitoring military targets, facilitating natural disaster relief, clinical bio-health monitoring, hazardous environment exploration, and seismology [3,4].

**Figure 1:**
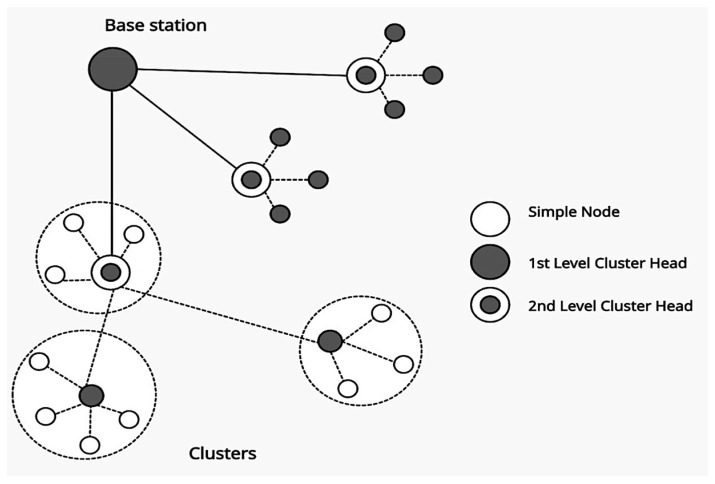
Hierarchical protocol structure.

An approach for regulating and conserving WS node resources is critical to maintaining proper operations. This priority has led to the proposal of several energy-consumption routing protocols for WSNs [4]. Hierarchical and cluster routing protocols (as shown in Figure 1) are notable for encouraging more energy-efficient operations, handling large amounts of data, and covering large areas without sacrificing service quality [5,6]. For example, a Low Energy Adaptive Clustering Hierarchy (LEACH) [7] is a fundamental energy-efficient routing protocol that allows networks to operate at their full capacity with minimum power consumption. The LEACH’s operation is based on the use of clusters, sensor nodes, and base stations (BSs). 

Each cluster has sensor nodes (SNs) that sense the environment and transmit the sensed data to a cluster head (CH). The CH is then in charge of relaying this information to the BS. However, the LEACH suffers from several flaws that negatively affect its performance. For example, the LEACH system does not use a predetermined selection of CHs, but rather randomly selects CHs to ensure that all participants have an equal chance of competing in the CH selection process. Moreover, chronological clusters broadcast data indefinitely, consume more resources, and elapse more rapidly. The LEACH process consists of three phases: setup, steady-state, and analysis. 

In 2021, Youssef et al. [8] suggested an improvement to the LEACH by introducing a new version of LEACH called LEACH-PRO. This enhanced protocol maximizes the lifetime of WSNs by determining the CHs, adopting a probabilistic approach that weighs two scales: minimum distance to the BS and maximum residual energy.

Since WSNs suffer from several security weaknesses that can be exploited by attackers, enhanced security is a crucial factor in preserving a secure WSN environment. For example, an adversary can easily compromise the network if there is poor authentication and a lack of secure communication. As reported in [7,8,9,10], WSNs can also be compromised through the SNs. Adversaries can hack or sabotage sensor nodes, giving them complete control over the nodes and consequently disrupting networks. For these reasons, many researchers have recently focused their attention on blockchain technology because of its unique characteristics, including decentralization, which leads to a higher level of complexity in terms of the network being compromised by an adversary and a high level of security. This technology is very useful when it comes to WSNs because of their vulnerability to many types of attacks.

### 1.1. Contributions

Our paper’s contributions are as follows:

- We propose a security protocol (SLEACH-PRO) based on blockchain technology to secure WSNs.
- Burrows-Abadi-Needham (BAN) logic and the Automated Validation of Internet Security Protocols and Applications (AVISPA) tool are used to prove the security analysis and properties of the proposed SLEACH-PRO protocol against man in the middle (MITM) and replay attacks.
- The SLEACH-PRO is evaluated and compared with other related protocols regarding computational cost.
- SLEACH-PRO relies on hierarchical routing protocols based on selecting the cluster heads to perform transmission and processing operations to provide power to the rest of the devices. In addition, the sensor nodes in the SLEACH-PRO protocol are built on a probabilistic mechanism that identifies CHs based on the highest residual power and lowest distance from the base station, resulting in more efficient energy use.

### 1.2. Organization 

This paper is divided into eight sections. Section 2 studies the recent related approaches adopted by other researchers to secure the WSN environment using blockchain techniques. The motivation and the current security issues of the WSNs are highlighted in Section 4. The methodology is detailed in Section 4 and the proposed SLEACH-PRO in Section 5. Informal and formal security analyses of SLEACH-PRO are performed using both Burrows–Abadi–Needham (BAN) logic and the Automated Validation of Internet Security Protocols and Applications (AVISPA) tool in Section 6. Next, Section 7 illustrates the performance analysis of the SLEACH-PRO compared to the recent well-known related security protocols. Finally, the paper is concluded in Section 8.

## 2. Related Work

This section highlights the most recent related work conducted for securing the WSN environment by incorporating blockchain technology. This technology has received considerable attention from researchers in designing a security protocol for networks. According to [11], blockchain technology uses a consensus and decentralized mechanism to maintain data integrity, which effectively prevents data from being tampered with when it is transferred. The current section investigates recent approaches in the literature in order to analyze the proposed techniques and their demands for addressing the security challenges and requirements in WSNs. These approaches are as follows:

Kumar et al. [12] proposed the selection of redirection-nodes utilizing double-hop neighbor strategies using the computational trust-metric rooted with trust-power supports and trust-redirection supports for each hop-node. Furthermore, information is kept secure within attribute levels. On the other hand, standalone blocks hold data for specific attributes, which facilitates the restriction of the same data for users accessing only one block. Varying levels of encryption take place on each attribute, which can only be decrypted using the hash functions within the blockchain. This method has proven to have a high security level when information is routed, and it performs well overall.

Aziz et al. [13] proposed a system that authenticates the nodes in WSNs with the help of blockchain technology. Due to the effect of nodes, when not authenticated in the routing processes, planning for the various paths becomes a complex issue. In addition, the work proposed registration with Certificate-Authority Nodes (CANs), which authenticates the nodes, allows storage of input data, and disallows unverified nodes from taking part in the network. Conversely, the CH is not as computationally capable as the BS, and it does not have as much capacity to store data. Moreover, the CH would be responsible for the processing of received information from the SN, which would then be sent to the BS. Here, a secure hash algorithm (SHA)-256 hash function is utilized for verification for registering nodes on the network. Furthermore, within this proposal, CHs use power and distance-related information to select the nodes which function as forwarders. Simulations have revealed faster packet delivery and longer network lives.

Yang et al. [14] suggested a novel scheme to route data with the help of artificial intelligence and improve how securely and efficiently data is routed within a WSN. Data is routed from each node within the blockchain, making it un-traceable and tamperproof. In the proposed work, reinforcement-learning models assist with the dynamic routing of nodes and define each link based on effectivity and reliability. Consequently, comparing this model to conventional systems shows that there is a great difference in terms of registration by the registry node after the networks have been accessed. Each packet is then forwarded using routing nodes before the succeeding phase. This is followed by the confirmation of the routed data, addresses of the following nodes, packet quantities, and timestamps within the block-chain. Moreover, consensus mechanisms from the blockchain are then used by the server node to facilitate the confirmation of the same data and update what is stored on the blockchain. The data is then pulled, and the following route policies are reported to the routing nodes. Analysis shows that the model can perform efficiently despite having a 50% threat of a malicious node. The model also consumes less power and is more productive compared to the feasibility and efficiency of other models.

Abd El-Moghith et al. [15] proposed a system that includes a deep blockchain and a Markov decision process (MDP) that secures the WSN and makes it more efficient. Nodes are authenticated using Proofs-of-Authority (PoAs) that run on the blockchain. The system uses artificial intelligence to identify the groups needed to validate audits. This system focuses on the distinctive features of a contract. An appropriate procedure is then formulated using the MDP to transfer data simply and securely. Simulations reveal that the framework is 50% more efficient than the systems currently used to route data and that it can systematically remove an attack by unverified nodes. The system response times are also found to be more than adequate.

Jerbi et al. [16] introduced a system proposal that includes CHs that authenticate each member node within WSNs and BSs. The proposal involves a mobile node moving between networks and collecting information about CHs or using relay nodes to form blocks with another node during rounds, usually LEACH. The simulation results prove that the system is secured, robust, resilient, and able to calculate data faster, and that it allows devices to send quantitative messages to BSs in a shorter period of time.

Awan et al. [17] used an identification storage system within their blockchain to authenticate nodes within the network. The SNs are already privately identified, but the CH nodes must be authenticated. This means that the system does not necessarily authenticate every entity within the WSN. An unauthenticated node would therefore still have access to every resource within the network, which indicates that the node could technically behave maliciously. This may cause increased packet delays and negatively affect their delivery rates. The system also indicates trust assessment mechanisms to facilitate and authenticate the nodes on the blockchain when data is routed within networks. Due to the constraints in terms of power available for computation, the SN senses data and transmits it to the appropriate CH, which then sends the data to the appropriate BS. The BS has the power to perform calculations and other resources available to process this data. The proposal discusses maintaining this system both locally and publicly on the blockchain, using BS and CH, respectively, and it improves package delivery rates and proportions.

Cui et al. [18] introduced a scheme that allows multiple authentications of Internet of Things (IoT) services based on the blockchain. Each node can be defined as either a BS, master, or system cluster depending on its function, forming a hierarchy within its framework. Each node is then connected to a network on the blockchain and forms a hybrid model, which includes local strings and public strings. In the scenario in which nodes communicate with each other to authenticate each other, the traditional methods of identification are performed locally, and the head nodes of the block are publicly identified. Contract validation takes place in four stages: it is configured based on the BS to con-figure each parameter through each subnet node that may remain secured. BSs are then registered, and the blockchain record of the same is kept for the public. Accordingly, the nodes are authenticated and appropriately given authentication and authorization to operate within the network. The final stage involves logging out of specific suspicious nodes to prevent malicious intent attacks and other damage, such as a depleted power supply. The simulation results show that this model is secure and works efficiently. A summary of the secured routing protocol utilizing blockchain in the WSN is shown in Table 1.

## 3. Motivation and Problem Statement

Since WSNs use insecure channels for data transmission, their communication suffers from significant security flaws. Therefore, authentication is crucial for maintaining successful communication in WSNs. However, despite the different existing authentication schemes in the literature [12,13,14,15,16,17,18] that significantly fulfill the required protection, the issue of greater performance remains challenging for researchers. Security enhancement and energy efficiency are essential to preserving resource availability, and fewer computational processes can drive better performance. Hence, several current WSN routing protocols have been investigated and analyzed, and the benefits of the LEACH-PRO explored for extending the lifetime of WSN nodes by employing energy-efficiency techniques. Designing an authentication routing protocol by deploying the blockchain with LEACH-PRO is vital in maximizing the network’s lifetime and maintaining secure communication that resists the threats to security in WSNs.

## 4. Methodology 

To overcome the security concerns and improve the performance of WSNs, a novel SLEACH-PRO is proposed for validating WSN entities and maintaining secure communication. In this model, the network’s entities are initially registered with BSs and obtain the CARD-ID required later for authentication. Thus, the proposed SLEACH-PRO protocol performs a decentralized authentication mechanism by applying a blockchain to multiple base stations to maintain data integrity and avoid system and performance degradation in the event of a station failure. Secure data exchange then takes place between the sensor and the end user in a secure manner using a shared key (SHK) generated by Elliptic Curve Cryptography (ECC) in the initialization phase. In the next stage, BAN logic and the AVISPA tool are used to prove the formal security analysis of the SLEACH-PRO and its security against known attacks in WSN environments. Then, the proposed protocol is evaluated and compared to related protocols in terms of computational cost and security level based on its resistance against several attacks.

## 5. Proposed SLEACH-PRO Protocol

This section introduces the network model and the sequential operation phases of the proposed SLEACH-PRO protocol.

### 5.1. Network Model 

**Figure 2:**
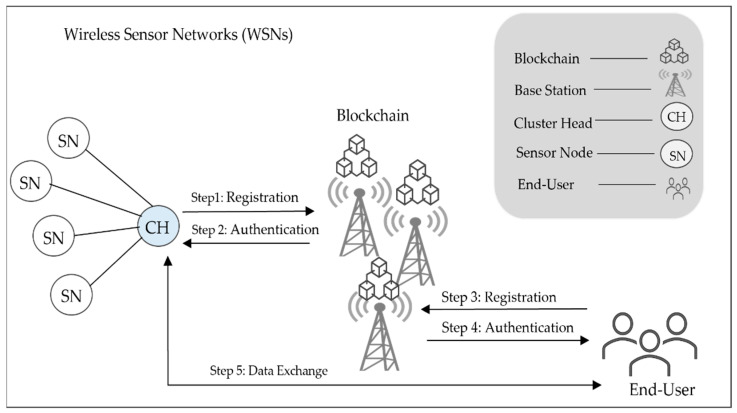
The framework of the proposed model.

Generally, WSNs consist of sensor nodes, cluster heads (CHs), and base stations (BSs) deployed in a geographical area to monitor the environment, as shown in Figure 2. Thus, a sensor node detects and monitors its surroundings and then sends the monitored data to the associated CH for relaying data to the BSs. The BSs then forward the received data to the user. Security is part of this communication by deploying the blockchain on BSs. To protect the network communication, four phases are considered: The first phase is the initialization, wherein the security parameters of the network nodes are initialized by the base station, and a shared key is generated by the elliptic curve algorithm. The second phase is the registration, in which the base station issues identification cards to network entities for later use in authentication. The third phase is authentication, where the smart contracts are used to authenticate all network entities to avoid malicious nodes. The last phase refers to the secure data exchange between the end user and the sensor using a shared key (SHK) to encrypt messages.

### 5.2. CH Selection Criteria

The SLEACH-PRO protocol has the same design logic as LEACH-PRO, selecting CHs using two criteria: minimum distance to the base and maximum residual energy. It works in a round-robin style, so LEACH-PRO is LEACH. The processes are divided into two phases during each round: preparation and steady-state. When the required parameters are met, the sensors located at the sensing field boundary are excluded from participating in the CH selection procedure [8].

### 5.3. Network Phases of the SLEACH-PRO Protocol

**Figure 3:**
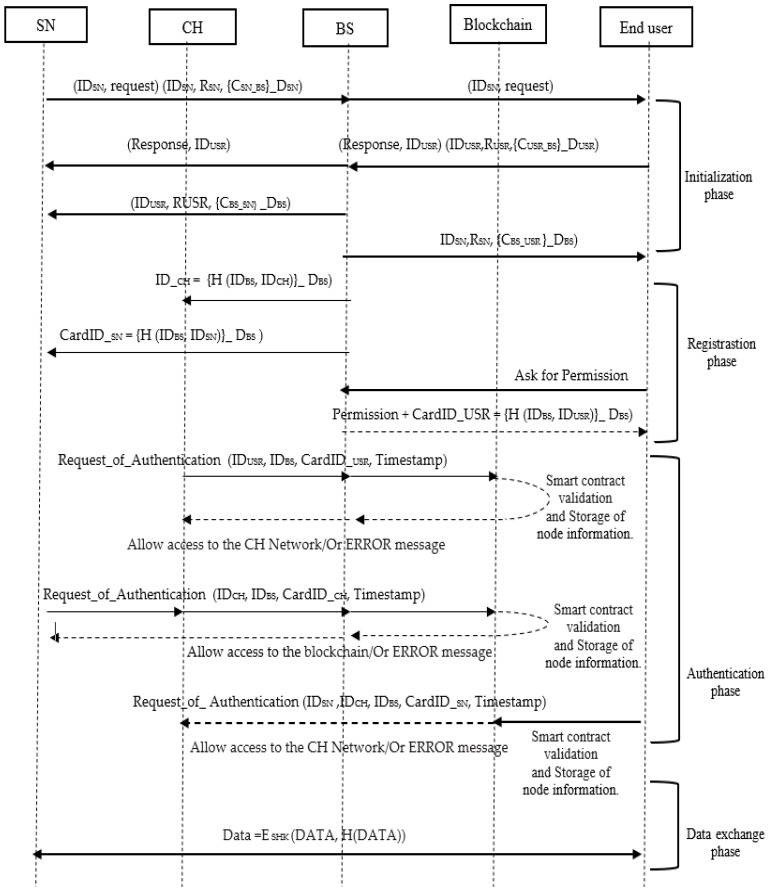
Overall flow of the SLEACH-PRO.

The SLEACH-PRO protocol consists of four phases: initialization, registration, authentication, and data exchange, as shown in Figure 3. The notations used in the proposed SLEACH-PRO protocol are listed below in Table 2.

#### 5.3.1. Node’s Initialization Phase

First, the BS initializes all nodes in the network before deploying them. To get the node’s unique identity, the BS computes an identification for each node, including itself (Mac address), and sends it to each node for storage. The sensor node’s identity is indicated as (ID_SN_), the CH node’s identity as (ID_CH_), the BS’s identity as (ID_BS_), and the end user’s identity as (ID_USR_). The system parameters are initialized and defined by the BS as follows:

- BS chooses a finite field F_q_ over q > 2^160^;
- BS chooses an elliptic curve Eq (a, b): y^2^ mod q = (x^3^ + ax + b) mod q with order n over Fq, where a, b € F and (4a^3^ + 27b^2^) ≠ 0 mod q;
- BS chooses a base point Q of order n over Eq (a, b);
- BS publishes Eq (a, b), Ek()/Dk() and Q;
- The SN and USR must register to BS to generate their private/public key pair *(d_SN_/U_SN_)* and *(d_USR_/U_USR_)*. The private/public key pair of BS is *(d_BS_/U_BS_)*, where U_SN_ = d_SN_.Q, U_USR_ = d_USR_.Q and U_BS_ =d_BS_.Q.

#### 5.3.2. Node’s Registration Phase

In this phase, the BS generates *CardIDs* for all nodes in the network, where their identity is verified. The *CardID* contains a hash function containing the identification of the *BS ID_BS_* of the WSN in which the nodes reside and defines the ID_CH_ of the CH node or the *ID_SN_* of the sensor node. The *CardID* is signed using the *BS’s* private key.

*CardID__CH_ = {H (ID_BS_, ID_CH_)}_D_BS_),*

*CardID__SN_ = {H (ID_BS_, ID_SN_)}_D_BS_)*

#### 5.3.3. Node’s Authentication Phase

A node identity information storage structure is proposed in this paper, which consists largely of the IP address, public key, BS identifier of a WSN node, and a CH node, which is the network identifier of the cluster network to which a node belongs.

##### Cluster Head

The CH then initiates the authentication process by sending a message to the BS and letting the blockchain know the results.

*Request_of_ Authentication (ID_CH_, ID_BS_, CardID__CH_, Timestamp)*

Until the smart contract performs the authentication verification process, it is run on the blockchain. The steps involved in the smart contract are outlined in the following steps:

- Verify the timestamp and its validity;
- By querying the identification information of a node in the blockchain, the CH verifies whether it already exists in the blockchain or not. If a node exists, the Verify function fails;
- Verify the *BS* identification *ID_BS_* and its validity;
- The smart contract verifies the CardID__CH_ and its validity using the *ID_BS_* and *ID_CH_* in the *Request_of_Authentication* transaction request message and verifies that the hash function is signed using the base station (*BS*)’s public key in the message. By calculating the hash value and comparing it with the message’s hash value, the smart contract determines whether the message hash is valid;

If any of the previous steps are unsuccessful, an error message is sent to the *BS*. As long as the validation process is successful, the blockchain stores the identity of the *CH* node and publishes the validated message. After that, the blockchain agrees not to allow the *CH* to access the network.

##### Sensor Node

Sensor nodes perform the authentication process on the blockchain. Each sensor node joins only one cluster network.

*Request_of_ Authentication (ID_SN_, ID_CH_, ID_BS_, CardID__SN_, Timestamp)*

The *CH*, when it receives the *Request_of_Authentication*, checks the timing of the timestamp. If the timing is correct, the user’s registration transaction will be sent to the blockchain network until they register their information. The authentication process takes place in the following steps:

- Verify the timestamp and its validity;
- By querying the identification information of a node in the blockchain, the sensor node verifies whether it already exists in the blockchain or not. If a node exists, the Verify function fails;
- Verify if the ID_CH_ CH node exists on the blockchain. The registration fails if *CH* doesn’t exist;
- Verify *BSn* identification *ID_BS_* and its validity;
- The smart contract verifies the CardID__SN_ and its validity using the *ID_BS_* and *ID_SN_* in the *Request_of_Authentication* transaction request message and verifies that the hash function is signed using the BS’s public key in the message. Smart contracts calculate their hash values and compare them with the hash values contained within messages.

When any of the previous verification steps fail, an error message is sent to the *CH*. Upon successful verification, the identity information for the sensor node will be uploaded to the blockchain, where it will be stored. The blockchain then agrees that the sensor nodes can access the *CH* network.

##### End User

The end user connects to the sensor node and authenticates with the BS. Users first must obtain their own identification certificates, which can be received from manufacturers, administrative departments, and other organizations. The end user must then get permission from the BS, which generates the CardID to the user. Then, the user sends an authentication request to the blockchain, and the end user’s identity is authenticated by running the smart contract, which provides a confirmation message to the user and the CH node via the blockchain. Lastly, the authentication credentials between the end user and the cluster’s header node are generated. The end user creates a secure connection with the sensor node after verification.

*CardID__USR_ = {H (ID_BS_, ID_USR_)}_D_BS_)*

The *USR* then initiates the authentication process by sending a message to the *BS* and letting the blockchain know the results.

*Request_of_ Authentication (ID_USR_, ID_BS_, CardID__USR_, Timestamp)*

Until the smart contract performs the authentication verification process, it is run on the blockchain. The steps involved in the smart contract are outlined in the following steps:

- Verify the timestamp and its validity;
- By querying the identification information of a node in the blockchain, the user verifies whether it already exists in the blockchain or not. If the user exists, the Verify function fails;
- Verify the BS identification *ID_BS_* and its validity;
- The smart contract verifies the *CardID__USR_* and its validity using the *ID_BS_* and *ID_USR_* in the *Request_of_ Authentication* transaction request message and verifies that the hash function is signed using the BS’s public key in the message. By calculating the hash value and comparing it with the message’s hash value, the smart contract determines whether the message hash is valid.

If any of the previous steps are unsuccessful, an error message is sent to the *BS*. As long as the validation process is successful, the blockchain stores the identity of the *USR* and publishes the validated message. After that, the blockchain agrees not to allow the CH to access the network.

#### 5.3.4. Data Exchange Phase

In the phase of exchanging data between the sensor and the end user, which data is encrypted using the shared key (*SHK*), *SHK* is based on the ECC.

*Data_Message = E_SHK_(DATA, H(DATA))*

## 6. Security Analysis of the SLEACH-PRO Protocol 

### 6.1. SLEACH-PRO Using BAN Logic

To eliminate the potential security flaws in the design process of the SLEACH-PRO protocol, Burrows-Abadi-Needham (BAN) logic [19] is used to analyze the authentication and verification processes that can be regarded as a good proof of correctness under the assumptions. Table 3 shows the notations used in BAN logic.

The BAN logic assumptions used in building the sequencing process for analyzing the SLEACH-PRO protocol are listed in Table 4. 

Since the SLEACH-PRO protocol has five stages, and each stage has several frames exchanged between the network entities, the security of the proposed protocol is analyzed and validated using BAN logic, as follows:

#### 6.1.1. Initialization Phase

In the initialization phase, the security parameter is exchanged, and communication is established between the nodes and the user. Message (1) is sent by the sensor to the BS for the exchange of the ECC security parameters to generate the shared key between sensor and user. The message contains ID_SN_, R_SN_, and a certificate containing H (ID_SN_, R_SN_, ECCP) signed by a sensor private key to ensure integrity. Message (2) is transmitted by the sensor to the user to establish the communication between them.

(1) $$\begin{array}{cc} \mathrm{SN}\to \;\mathrm{BS} & \;\;\mathrm{SN}\;I\sim \;\{{\mathrm{ID}}_{\mathrm{SN}},\;R_{\mathrm{SN}},\;\{H\;({\mathrm{ID}}_{\mathrm{SN}},\;R_{\mathrm{SN}},\;\mathrm{ECCP})\;K_{\mathrm{SN}}^{-1}\}\\ & \;\;\mathrm{BS}\;⊲\;\{{\mathrm{ID}}_{\mathrm{SN}},\;R_{\mathrm{SN}},\;\{H\;({\mathrm{ID}}_{\mathrm{SN}},\;R_{\mathrm{SN}},\;\mathrm{ECCP})\;K_{\mathrm{SN}}^{-1}\}\\ & \;\;\mathrm{BS}\;⊲\;\{{\mathrm{ID}}_{\mathrm{SN}},\;R_{\mathrm{SN}},\;\{H\;({\mathrm{ID}}_{\mathrm{SN}},\;R_{\mathrm{SN}},\;\mathrm{ECCP})\;K_{\mathrm{SN}}^{-1}\}\overset{K}{\to }{}_{\mathrm{SN}}\\ & \;\;\{{\mathrm{ID}}_{\mathrm{SN}},\;R_{\mathrm{SN}},\;H\;({\mathrm{ID}}_{\mathrm{SN}},\;R_{\mathrm{SN}},\;\mathrm{ECCP})\} \end{array}$$

(2) $$\begin{array}{cc} \mathrm{SN}\;\to \;\mathrm{USR} & \;\;\mathrm{SN}I\sim \{{\mathrm{ID}}_{\mathrm{SN}},\mathrm{Request}\}\\ & \;\;\mathrm{USR}⊲\{{\mathrm{ID}}_{\mathrm{SN}},\mathrm{Request}\}\\ & \;\;\{{\mathrm{ID}}_{\mathrm{SN}},\mathrm{Request})\} \end{array}$$

Message (3) is sent by the user to the BS to exchange the ECC security parameters to generate the shared key between sensor and user. The message contains ID_USR_, R_USR_, and a certificate containing H (ID_USR_, R_USR_, ECCP) signed by a user private key to ensure the integrity. Message (4) is transferred from the user to the sensor to establish the communication between them.

(3) $$\begin{array}{cc} \mathrm{USR}\to \;\mathrm{BS} & \;\;\mathrm{USR}\;I\sim \;\{{\mathrm{ID}}_{\mathrm{USR}},\;R_{\mathrm{USR}},\;\{H\;({\mathrm{ID}}_{\mathrm{USR}},\;R_{\mathrm{USR}},\;\mathrm{ECCP})\;K_{\mathrm{USR}}^{-1}\}\\ & \;\;\mathrm{BS}\;⊲\;\{{\mathrm{ID}}_{\mathrm{USR}},\;R_{\mathrm{USR}},\;\{H\;({\mathrm{ID}}_{\mathrm{USR}},\;R_{\mathrm{USR}},\;\mathrm{ECCP})\;K_{\mathrm{USR}}^{-1}\}\\ & \;\;\mathrm{BS}⊲\;\{{\mathrm{ID}}_{\mathrm{USR}},\;R_{\mathrm{USR}},\;\{H\;({\mathrm{ID}}_{\mathrm{USR}},\;R_{\mathrm{USR}},\;\mathrm{ECCP})\;K_{\mathrm{USR}}^{-1}\}\overset{K}{\to }{}_{\mathrm{USR}}\\ & \;\;\{{\mathrm{ID}}_{\mathrm{USR}},\;R_{\mathrm{USR}},\;\{H\;({\mathrm{ID}}_{\mathrm{USR}},\;R_{\mathrm{USR}},\;\mathrm{ECCP})\} \end{array}$$

(4) $$\begin{array}{cc} \mathrm{USR}\to \;\mathrm{SN} & \;\;\mathrm{USR}I\sim \;\{{\mathrm{ID}}_{\mathrm{SN}},\;\mathrm{Response}\}\\ & \;\;\mathrm{SN}⊲\{{\mathrm{ID}}_{\mathrm{SN}},\;\mathrm{Response}\}\\ & \;\;\{{\mathrm{ID}}_{\mathrm{SN}},\;\mathrm{Response}\} \end{array}$$

Message (5) is delivered from the BS to the sensor to exchange the ECC security parameters to generate the shared key between sensor and user. The message contains R_USR_, and a certificate containing H (ID_USR_, R_USR_, ECCP) signed by a BS private key to ensure the message’s authenticity.

(5) $$\begin{array}{cc} \mathrm{BS}\to \mathrm{SN} & \;\;\mathrm{BS}\;I\sim \;\{R_{\mathrm{USR}},\;\{H\;({\mathrm{ID}}_{\mathrm{USR}},\;R_{\mathrm{USR}},\;\mathrm{ECCP})\;K_{\mathrm{BS}}^{-1}\}\\ & \;\;\mathrm{SN}\;⊲\;\{R_{\mathrm{USR}},\;\{H\;({\mathrm{ID}}_{\mathrm{USR}},\;R_{\mathrm{USR}},\;\mathrm{ECCP})\;K_{\mathrm{BS}}^{-1}\}\\ & \;\;\mathrm{SN}⊲\;\{R_{\mathrm{USR}},\;\{H\;({\mathrm{ID}}_{\mathrm{USR}},\;R_{\mathrm{USR}},\;\mathrm{ECCP})\;K_{\mathrm{BS}}^{-1}\}\overset{K}{\to }{}_{\mathrm{BS}}\\ & \;\;\{R_{\mathrm{USR}},\;\{H\;({\mathrm{ID}}_{\mathrm{USR}},\;R_{\mathrm{USR}},\;\mathrm{ECCP})\} \end{array}$$

Message (6) is sent by the BS to the user for the exchange of the ECC security parameters to generate the shared key between sensor and user. The message contains R_SN_ and a certificate containing H (ID_SN_, R_SN_, ECCP) signed by a BS private key to ensure the message’s authenticity.

(6) $$\begin{array}{cc} \mathrm{BS}\to \mathrm{USR} & \;\;\mathrm{BS}\;I\sim \;\{R_{\mathrm{SN}},\;\{H\;({\mathrm{ID}}_{\mathrm{SN}},\;R_{\mathrm{SN}},\;\mathrm{ECCP})\;K_{\mathrm{BS}}^{-1}\}\\ & \;\;\mathrm{USR}\;⊲\;\{R_{\mathrm{SN}},\;\{H\;({\mathrm{ID}}_{\mathrm{SN}},\;R_{\mathrm{SN}},\;\mathrm{ECCP})\;K_{\mathrm{BS}}^{-1}\}\\ & \;\;\mathrm{USR}\;⊲\;\{R_{\mathrm{SN}},\;\{H\;({\mathrm{ID}}_{\mathrm{SN}},\;R_{\mathrm{SN}},\;\mathrm{ECCP})\;K_{\mathrm{BS}}^{-1}\}\overset{K}{\to }{}_{\mathrm{BS}}\\ & \;\;\{R_{\mathrm{SN}},\;\{H\;({\mathrm{ID}}_{\mathrm{SN}},\;R_{\mathrm{SN}},\;\mathrm{ECCP})\} \end{array}$$

#### 6.1.2. Registration Phase

This phase analyzes the registration phase of the entities in the network from messages (7, 8, and 9) between the nodes and the ground station. Message (7) explains the card identification message used for authentication, which is sent from the BS to the CH. The message includes ID_BS_ and H (ID_BS_, ID_CH_) signed by a BS private key to ensure the message’s authenticity.

(7) $$\begin{array}{cc} \mathrm{BS}\to \;\mathrm{CH} & \;\;\mathrm{BS}I\sim \{{\mathrm{ID}}_{\mathrm{BS}},\{H({\mathrm{ID}}_{\mathrm{BS}},{\mathrm{ID}}_{\mathrm{CH}})K_{\mathrm{BS}}^{-1}\}\\ & \;\;\mathrm{CH}\;⊲\{{\mathrm{ID}}_{\mathrm{BS}},\;\{H({\mathrm{ID}}_{\mathrm{BS}},\;{\mathrm{ID}}_{\mathrm{CH}})\;K_{\mathrm{BS}}^{-1}\}\\ & \;\;\mathrm{CH}\;⊲\{{\mathrm{ID}}_{\mathrm{BS}},\;\{H({\mathrm{ID}}_{\mathrm{BS}},\;{\mathrm{ID}}_{\mathrm{CH}})\;K_{\mathrm{BS}}^{-1}\}\overset{K}{\to }{}_{\mathrm{BS}}\\ & \;\;\{{\mathrm{ID}}_{\mathrm{BS}},\;\{H({\mathrm{ID}}_{\mathrm{BS}},\;{\mathrm{ID}}_{\mathrm{CH}})\;\} \end{array}$$

Message (8) includes the card identification message sent from the BS to a sensor for authentication. The message also includes ID_BS_ and H (ID_BS_, ID_SN_) signed by a BS private key to ensure the authenticity of the message.

(8) $$\begin{array}{cc} \mathrm{BS}\;\to \;\mathrm{SN} & \;\;\mathrm{BS}\;I\sim \{{\mathrm{ID}}_{\mathrm{BS}},\;\{H({\mathrm{ID}}_{\mathrm{BS}},\;{\mathrm{ID}}_{\mathrm{SN}})\;K_{\mathrm{BS}}^{-1}\}\\ & \;\;\mathrm{SN}\;⊲\{{\mathrm{ID}}_{\mathrm{BS}},\;\{H({\mathrm{ID}}_{\mathrm{BS}},\;{\mathrm{ID}}_{\mathrm{SN}})\;K_{\mathrm{BS}}^{-1}\}\\ & \;\;\mathrm{SN}\;⊲\{{\mathrm{ID}}_{\mathrm{BS}},\;\{H({\mathrm{ID}}_{\mathrm{BS}},\;{\mathrm{ID}}_{\mathrm{SN}})\;K_{\mathrm{BS}}^{-1}\}\overset{K}{\to }{}_{\mathrm{BS}}\\ & \;\;{\mathrm{ID}}_{\mathrm{BS}},\;\{H({\mathrm{ID}}_{\mathrm{BS}},\;{\mathrm{ID}}_{\mathrm{SN}})\} \end{array}$$

Message (9) explains the card identification message sent from the BS to the user that is used for authentication. The message includes ID_BS_ and H (ID_USR_, ID_BS_) signed by a BS private key to ensure message’s integrity

(9) $$\begin{array}{cc} \mathrm{BS}\to \mathrm{USR} & \;\;\mathrm{BS}\;I\sim \{{\mathrm{ID}}_{\mathrm{BS}},\;\{H({\mathrm{ID}}_{\mathrm{BS}},\;{\mathrm{ID}}_{\mathrm{USR}})\;K_{\mathrm{BS}}^{-1}\}\\ & \;\;\mathrm{USR}\;⊲\{{\mathrm{ID}}_{\mathrm{BS}},\;\{H({\mathrm{ID}}_{\mathrm{BS}},\;{\mathrm{ID}}_{\mathrm{USR}})\;K_{\mathrm{BS}}^{-1}\}\\ & \;\;\mathrm{USR}\;⊲\{{\mathrm{ID}}_{\mathrm{BS}},\;\{H({\mathrm{ID}}_{\mathrm{BS}},\;{\mathrm{ID}}_{\mathrm{USR}})\;K_{\mathrm{BS}}^{-1}\}\overset{K}{\to }{}_{\mathrm{BS}}\}\\ & \;\;\{{\mathrm{ID}}_{\mathrm{BS}},\;\{H({\mathrm{ID}}_{\mathrm{BS}},\;{\mathrm{ID}}_{\mathrm{USR}})\}\} \end{array}$$

#### 6.1.3. Authentication Phase

In this phase, the nodes’ information is verified by the blockchain applied in the BS, and the authentication is completed. Message (10) refers to the request message for authentication sent by the CH to the BS. The message contains (ID_BS_, ID_CH_, and the card identification, which is a hash of the ID_BS_, ID_CH_ (H (ID_CH_, ID_BS_)) signed by the BS’s private key to ensure integrity and timestamp. The validation is complete after verifying the information of the CH through the smart contract.

(10) $$\begin{array}{cc} \mathrm{CH}\to \;\mathrm{BS} & \;\;\mathrm{CH}\;I\sim \{{\mathrm{ID}}_{\mathrm{BS}},\;{\mathrm{ID}}_{\mathrm{CH}},\;\{H({\mathrm{ID}}_{\mathrm{BS}},\;{\mathrm{ID}}_{\mathrm{CH}})\;K_{\mathrm{BS}}^{-1}\},\;T_{N}\}\\ & \;\;\mathrm{BS}\;⊲\{{\mathrm{ID}}_{\mathrm{BS}},\;{\mathrm{ID}}_{\mathrm{CH}},\;\{H({\mathrm{ID}}_{\mathrm{BS}},\;{\mathrm{ID}}_{\mathrm{CH}})\;K_{\mathrm{BS}}^{-1}\},\;T_{N}\}\\ & \;\;\mathrm{BS}\;⊲\{{\mathrm{ID}}_{\mathrm{BS}},\;{\mathrm{ID}}_{\mathrm{CH}},\;\{H({\mathrm{ID}}_{\mathrm{BS}},\;{\mathrm{ID}}_{\mathrm{CH}})\;K_{\mathrm{BS}}^{-1}\},\;T_{N}\}\overset{K}{\to }{}_{\mathrm{BS}}\\ & \;\;\{{\mathrm{ID}}_{\mathrm{BS}},\;{\mathrm{ID}}_{\mathrm{CH}},\;\{H({\mathrm{ID}}_{\mathrm{BS}},\;{\mathrm{ID}}_{\mathrm{CH}})\},\;T_{N}\} \end{array}$$

Messages (11 and 12) explain the request message for authentication sent from the sensor to the cluster head and forwarded to the BS. The message contains (ID_BS_, ID_CH_, ID_SN_, and the card identification containing H (ID_BS_, ID_SN_)) signed by a BS private key to ensure integrity and timestamp. The validation is complete after verifying the information of the SN through the smart contract.

(11) $$\begin{array}{cc} \mathrm{SN}\to \mathrm{CH} & \;\;\mathrm{SN}\;I\sim \{{\mathrm{ID}}_{\mathrm{SN}},\;{\mathrm{ID}}_{\mathrm{BS}},\;{\mathrm{ID}}_{\mathrm{CH}},\;\{H({\mathrm{ID}}_{\mathrm{BS}},\;{\mathrm{ID}}_{\mathrm{SN}})\;K_{\mathrm{BS}}^{-1}\},\;T_{N}\}\\ & \;\;\mathrm{CH}\;⊲\{{\mathrm{ID}}_{\mathrm{SN}},\;{\mathrm{ID}}_{\mathrm{BS}},\;{\mathrm{ID}}_{\mathrm{CH}},\;\{H({\mathrm{ID}}_{\mathrm{BS}},\;{\mathrm{ID}}_{\mathrm{SN}})\;K_{\mathrm{BS}}^{-1}\},\;T_{N}\}\\ & \;\;\mathrm{CH}\;⊲\{{\mathrm{ID}}_{\mathrm{SN}},\;{\mathrm{ID}}_{\mathrm{BS}},\;{\mathrm{ID}}_{\mathrm{CH}},\;\{H({\mathrm{ID}}_{\mathrm{BS}},\;{\mathrm{ID}}_{\mathrm{SN}})\;K_{\mathrm{BS}}^{-1}\},\;T_{N}\}\overset{K}{\to }{}_{\mathrm{BS}}\\ & \;\;({\mathrm{ID}}_{\mathrm{SN}},\;{\mathrm{ID}}_{\mathrm{BS}},\;{\mathrm{ID}}_{\mathrm{CH}},\;\{H({\mathrm{ID}}_{\mathrm{BS}},\;{\mathrm{ID}}_{\mathrm{SN}})\},\;T_{N}\} \end{array}$$

(12) $$\begin{array}{cc} \mathrm{CH}\to \mathrm{BS} & \;\;\mathrm{SN}\;I\sim \{{\mathrm{ID}}_{\mathrm{SN}},\;{\mathrm{ID}}_{\mathrm{BS}},\;{\mathrm{ID}}_{\mathrm{CH}},\;\{H({\mathrm{ID}}_{\mathrm{BS}},\;{\mathrm{ID}}_{\mathrm{SN}})\;K_{\mathrm{BS}}^{-1}\},\;T_{N}\}\\ & \;\;\mathrm{BS}\;⊲\{{\mathrm{ID}}_{\mathrm{SN}},\;{\mathrm{ID}}_{\mathrm{BS}},\;{\mathrm{ID}}_{\mathrm{CH}},\;\{H({\mathrm{ID}}_{\mathrm{BS}},\;{\mathrm{ID}}_{\mathrm{SN}})\;K_{\mathrm{BS}}^{-1}\},\;T_{N}\}\\ & \;\;\mathrm{BS}\;⊲\{{\mathrm{ID}}_{\mathrm{SN}},\;{\mathrm{ID}}_{\mathrm{BS}},\;{\mathrm{ID}}_{\mathrm{CH}},\;\{H({\mathrm{ID}}_{\mathrm{BS}},\;{\mathrm{ID}}_{\mathrm{SN}})\;K_{\mathrm{BS}}^{-1}\},\;T_{N}\}\overset{K}{\to }{}_{\mathrm{BS}}\\ & \;\;\{{\mathrm{ID}}_{\mathrm{SN}},\;{\mathrm{ID}}_{\mathrm{BS}},\;{\mathrm{ID}}_{\mathrm{CH}},\;\{H({\mathrm{ID}}_{\mathrm{BS}},\;{\mathrm{ID}}_{\mathrm{SN}})\},\;T_{N}\} \end{array}$$

Message (13) is the request message for authentication sent from the user to the BS. The message includes (ID_BS_, ID_CH_, ID_USR_, and a hash generated from (ID_BS_ and ID_USR_). The hash is signed using the BS’s private key to ensure integrity and timestamp. The validation is complete after verifying the information of the USR through the smart contract.

(13) $$\begin{array}{cc} \mathrm{USR}\to \mathrm{BS} & \;\;\mathrm{USR}I\sim \{{\mathrm{ID}}_{\mathrm{USR}},{\mathrm{ID}}_{\mathrm{BS}},{\mathrm{ID}}_{\mathrm{CH}},\{H({\mathrm{ID}}_{\mathrm{BS}},{\mathrm{ID}}_{\mathrm{USR}})K_{\mathrm{BS}}^{-1}\},T_{N}\}\\ & \;\;\mathrm{BS}\;⊲\{{\mathrm{ID}}_{\mathrm{USR}},\;{\mathrm{ID}}_{\mathrm{BS}},\;{\mathrm{ID}}_{\mathrm{CH}},\;\{H({\mathrm{ID}}_{\mathrm{BS}},\;{\mathrm{ID}}_{\mathrm{USR}})\;K_{\mathrm{BS}}^{-1}\},\;T_{N}\}\\ & \;\;\mathrm{BS}\;⊲\{{\mathrm{ID}}_{\mathrm{USR}},\;{\mathrm{ID}}_{\mathrm{BS}},\;{\mathrm{ID}}_{\mathrm{CH}},\;\{H({\mathrm{ID}}_{\mathrm{BS}},\;{\mathrm{ID}}_{\mathrm{USR}})\;K_{\mathrm{BS}}^{-1}\},\;T_{N}\}\overset{K}{\to }{}_{\mathrm{BS}}\\ & \;\;\{{\mathrm{ID}}_{\mathrm{USR}},\;{\mathrm{ID}}_{\mathrm{BS}},\;{\mathrm{ID}}_{\mathrm{CH}},\;\{H({\mathrm{ID}}_{\mathrm{BS}},\;{\mathrm{ID}}_{\mathrm{USR}})\},\;T_{N}\} \end{array}$$

#### 6.1.4. Data Exchange Phase

In this phase, the message (14) indicates the process of exchanging data between the sensor node and the user. Both the hash of data H (data) and the data itself are delivered over a secure connection where the data are encrypted using the session key generated from the ECC in phase 1.

(14) $$\begin{array}{cc} \mathrm{SN}\to \mathrm{US} & \;\;\mathrm{SN}\;I\sim \{(\mathrm{Data}),\;(H\;(\mathrm{Data}\}\;{}_{\mathrm{SN}}\;{⇋}^{k}{}_{\mathrm{US}}\\ & \;\;\mathrm{US}\;⊲\{(\mathrm{Data}),\;(H\;(\mathrm{Data}\}\;{}_{\mathrm{SN}}\;{⇋}^{k}\;{}_{\mathrm{US}}\\ & \;\;\mathrm{US}\;⊲\{(\mathrm{Data}),\;(H\;(\mathrm{Data}\}\;{}_{\mathrm{SN}}\;{⇋}^{k}\;{}_{\mathrm{US}}\}\;{}_{\mathrm{SN}}\;{⇋}^{k}\;{}_{\mathrm{US}}\\ & \;\;\{(\mathrm{Data}),\;(H\;(\mathrm{Data}\} \end{array}$$

### 6.2. Formal Security Analysis Using AVISPA

**Figure 4:**
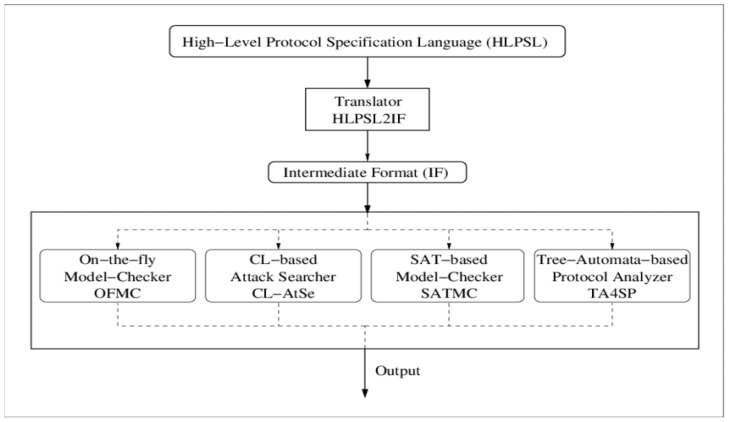
The AVISPA tool’s architecture [21].

With the proliferation of Internet-based services and the number of new security protocols being developed, the ability of humans to rigorously evaluate and validate them is outpaced. To facilitate the development of security protocols for the next generation as well as to improve their security, researchers need to have tools that enable a thorough study of these protocols by either detecting weaknesses or verifying their accuracy. To boost the speed and quality of protocol development and standardization processes, these tools should ideally be fully automated, robust, expressive, and easy to use. Having accurate information about a network is extremely important to the Internet security community. The AVISPA tool is a push-button automation tool that enables the computer to analyze Internet security-sensitive protocols and applications systematically by providing a modular and expressive formal language, which enables us to specify it in a manner that is as expressive as possible, and by integrating different back-end implementations that include an array of automatic methods that go start with protocol falsification. Figure 4 illustrates the integration between the current version of the tool and the four back-ends [20].

The security features of the proposed SLEACH-PRO are analyzed using AVISPA.

#### 6.2.1. Proposed SLEACH-PRO Protocol

A blockchain authentication system and the ECC algorithm were used to design the security protocol. One-way hashes are also included in the proposed protocol. As indicated in Table 4, the notations used in the proposed SLEACH-PRO protocol are listed as follows. A protocol normally consists of four phases: initialization, registration, authentication, and data exchange. Nodes are usually activated at the beginning of each phase.

In the initialization phase, the sensor and the user want to create a secure session key between them. The BS helps SNs and USRs authenticate each other via a public network. The following points show the details of how our protocol works.

- The SN chooses an integer r_SN_ ∈ Zq at random, calculates H_SN_ = H (r_SN_ ‘|| d_SN_) and R_SN_ = H_SN_.Q, and then calculates the security parameter K_SN_ = d_SN._ U_BS_ = d_SN_. d_BS_.Q, and the certificate C_SN_BS_ = H (ID_SN_ || R_SN_ || K_SN_). Next, the sensor sends the message (ID_SN_, request) and sends the certificate after it is signed with its private key (ID_SN_, R_SN_, {C_SN_BS_}_D_SN_) to the BS and USR.
- The USR receives the request message from the sensor (Request, ID_SN_), selects a random number r_USR_ ∈ Z_q_, and then computes H_USR_ = H (r_USR_’||d_USR_) and R_USR_ = H_USR_.Q. Then, the USR computes the security parameter K_USR_ = d_USR_·U_BS_ = d_USR_·d_BS_Q and the Certificate C_USR_BS_= H(ID_USR_||R_USR_||K_USR_). The user sends the message (ID_USR_, Response) and sends the certificate after it is signed with its private key (ID_USR_, R_USR_, {C_USR_BS_}_D_USR_) to the BS and SN.
- When the BS receives the messages (ID_SN_, R_SN_, {C_SN_BS_}_D_SN_) and (ID_USR_, R_USR_, {C_USR_BS_ }_D_USR_) from the SN and USR, it calculates the security parameters K_SNN_ = d_BS_.U_SN_ = d_SN_.d_BS_ .Q and K_USRR_ = d_BS_.U_USR_ = d_USR_.d_BS_.Q. After that, it computes ¯C_SN_BS_= H(ID_SN_||R_SN_||K_SNN_) using the R_SN_ in the message received from the SN and K_SNN_ computed by the BS. The BS then validates the condition ¯C_SN_BS =_? C_SN_BS_. The USR receives a failure-of-authentication message if the values are not equal. Otherwise, the BS computes C_BS_SN_ = H(ID_USR_||R_USR_||K_SNN_) and sends the certificate after it is signed with its private key (ID_USR_, R_USR_, {C_BS_SN_}_D_BS_) to the SN. Thereafter, the BS computes ¯C_USR_BS_ = H (ID_USR_||R_USR_||K_USRR_) using the R_USR_ in the message received from the USR and the K_USRR_ computed by the BS. The BS validates the condition ¯C_USR_BS =_? C_USR_BS_. If it is not equal, the BS notifies the SN that the authentication failed. Otherwise, the BS computes C_BS_USR_ = H (ID_SN_||R_SN_||K_USRR_) and sends the certificate after it is signed with its private key (ID_SN_, R_SN_, {C_BS_USR_}_D_BS_) to the USR.
- When the sensor receives the message (ID_USR_, R_USR_, {C_BS_SN_}_D_BS_), the SN computes C_BS_SN_ = H (ID_USR_||R_USR_||K_SNN_) by using his own R_SN_ and K_SN_ that was previously generated and the R_USR_ in the received message. After that, the SN validates the condition ¯C_BS_SN_ =? C_BS_SN_. If they are equal, the SN computes the shared key SHK = H (ID_SN_||ID_USR_||R_SN_||R_USR_||K), where the security parameter K = H_SN_·R_USR_ = H_SN_·H_USR_·Q. Otherwise, the SN closes the session. When the user receives the message (ID_SN_,R_SN_, {C_BS_USR_}_D_BS_), the USR computes ¯C_BS_USR_ = H(ID_SN_||R_SN_||K_USRR_) by using their own R_USR_ and K_USR_ that was previously generated and the R_SN_ in the received message. After that, the USR validates the condition ¯C_BS_USR_ =? C_BS_OSR_. If they are equal, the USR computes the shared key SHK = H (ID_SN_||ID_USR_||R_SN_||R_USR_||K), where the security parameter K = H_USR_·R_SN_ = H_USR_·H_SN_·Q. Otherwise, the USR closes the session.

**Figure 5:**
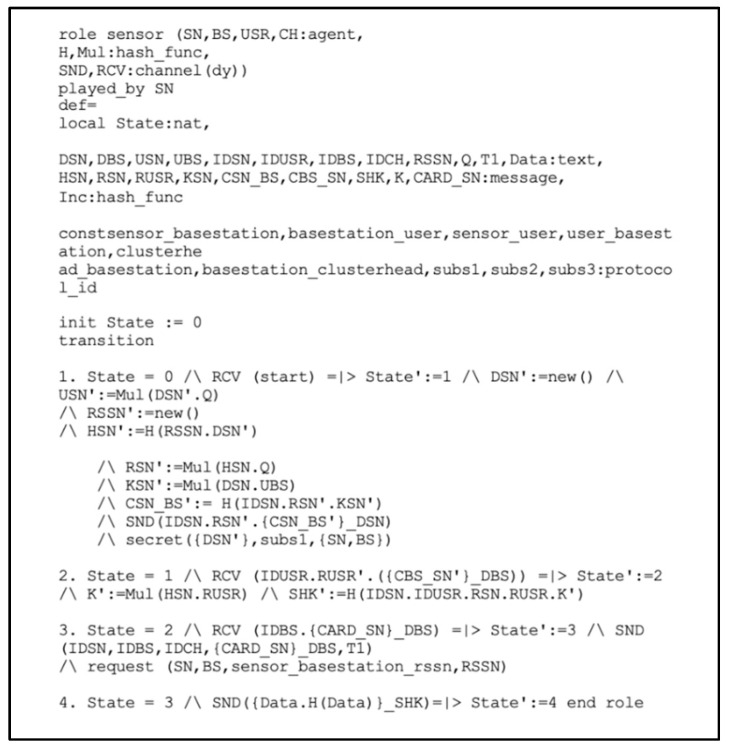
Role specification for sensor node in high-level protocol specification language (HLPSL).

In Figure 5, we implement the SN’s role. Initially, the SN sends (ID_SN_.R_SN_. {C_SN_BS_}_D_SN_) to the BS through an open channel. It is to be noted that the random number R_SNN_ was generated using a new () operation, and the SN transmits any message with the help of the Snd () operation. The declaration secret ({D_SN_}, subs1, (SN, BS)) specifies that the private key D_SN_ is only known to (SN, BS). 

In transition 2, A uses the Rcv() operation to receive (ID_USR_.R_USR_.{C_BS_SN_}_D_BS_) from the BS over an open channel, and then computes the shared key *SHRK = (ID_SN_.ID_USR_.R_SN_.R_USR_.K’)*. Then, the SN receives the message (ID_BS_,{cardID__SN_}_D_BS_) where the Card_ID consists of H (ID_SN_.ID_BS_)_D_BS,_ which is used in the authentication process by the smart contract applied to the BS. Next, the SN sends a Request_of_Authentication (ID_SN_, ID_CH_, BS_ID_, {CardID__SN_}_D_BS_, T_1_) and requests (SN, BS, sensor BS, RSSN) messages to perform the registration authentication process by running the smart contract applied to the BS. Finally, the SN sends a message (DATA, H(Data)_SHK) to the USR, which is encrypted with their shared key.

**Figure 6:**
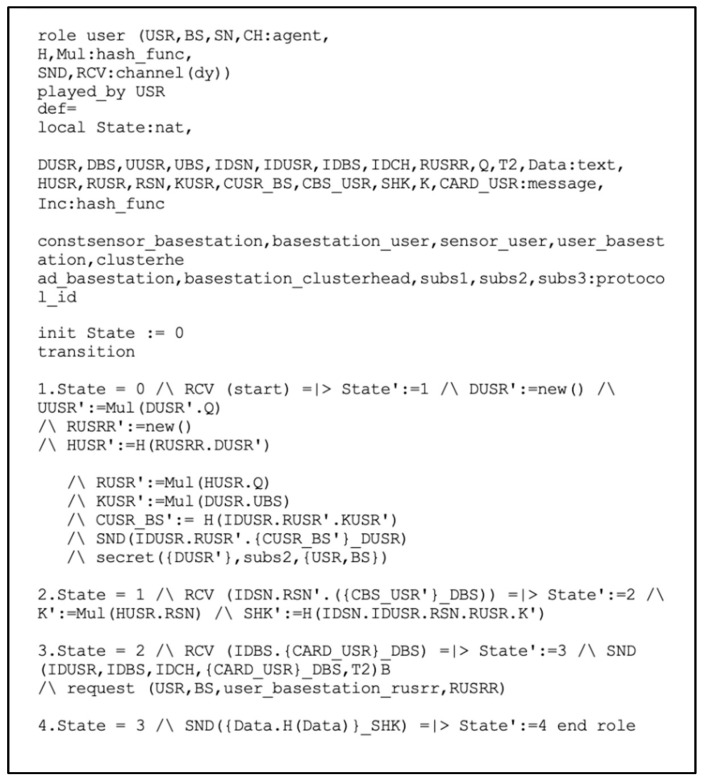
Role specification for the user in HLPSL.

Figure 6 illustrates the implementation of the USR’s role. Initially, the USR sends (ID_USR_. R_USR_. {C_USR_BS_}_D_USR_) to the BS through an open channel. It is to be noted that the random number R_USRR_ was generated using a new() operation, and the SN transmits any message with the help of the Snd() operation. The declaration secret ({D_USR_}, subs2, (USR, BS)) specifies that the private key D_USR_ is only known to (USR, BS). In transition 2, A uses the Rcv() operation to receive (ID_SN_.R_SN_.{C_BS_USR_}_D_BS_) from the BS over an open channel and then computes the shared key SHRK = (ID_SN_.ID_USR_.R_SN_.R_USR_.K’). Then, the USR receives the message (ID_BS_,{CardID__USR_}_D_BS_), where the Card_ID consists of H (ID_USR_.ID_BS_)_D_BS,_ which is used in the authentication process by the smart contract applied to the BS. Next, the SN sends a Request_of_Authentication (ID_USR_, ID_CH_, ID_BS_, {CardID__USR_}_D_BS_, T_2_) and request (USR, BS, usr_basestaion_r_USRR_, R_USRR_) messages to perform the registration authentication process by running the smart contract applied to the BS. Finally, the USR receives a message (DATA, H(Data)_SHK) from the SN, which is encrypted with their shared key.

**Figure 7:**
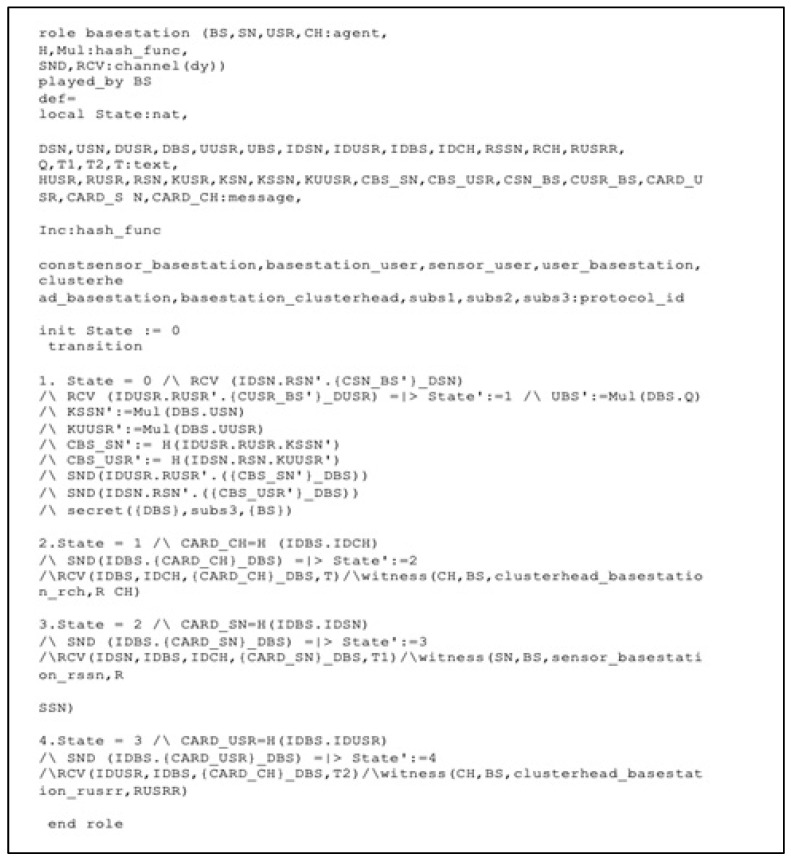
Role specification for the BS in HLPSL.

We illustrate the implementation of the role of the BS in the HLPSL language in Figure 7. The BS initially receives the messages (ID_SN_.R_SN_.{C_SN_BS_}_D_SN_) and (ID_USR_.R_USR_.{C_USR_BS_}_D_USR_) from the SN and USR in parallel. Then, the BS sends (R_USR_.{C_BS_SN_}_D_BS_) to the SN and (R_SN_.{C_BS_USR_}_D_BS_) to the USR, respectively. The declaration secret ({D_BS_}, subs3,{BS} means that the private key D_BS_ is kept secret indefinitely and is only known by the BS. Then, the BS generates a card ID for each network entity to use in the authentication process. Next, the BS sends a message (ID_BS_,{CardID__CH_}___D_BS_) to the CH (ID_BS_,{CardID__SN_}___D_BS_), to the SN (ID_BS_,{CardID__USR_}___D_BS_), and to the USR. After that, the BS receives authentication requests messages (ID_CH_, ID_BS_, {CardID__CH_}_D_BS_, T) from the CH, (ID_SN_,ID_CH_,ID_BS_, {CardID__SN_}_D_BS_, T_1_), the SN (ID_USR_, ID_CH_, ID_BS_, {CardID__USR_}_D_BS_, T_2_), and the USR. The authentication process is done by running the smart contract that was mentioned in Section 4, including how it works in detail.

**Figure 8:**
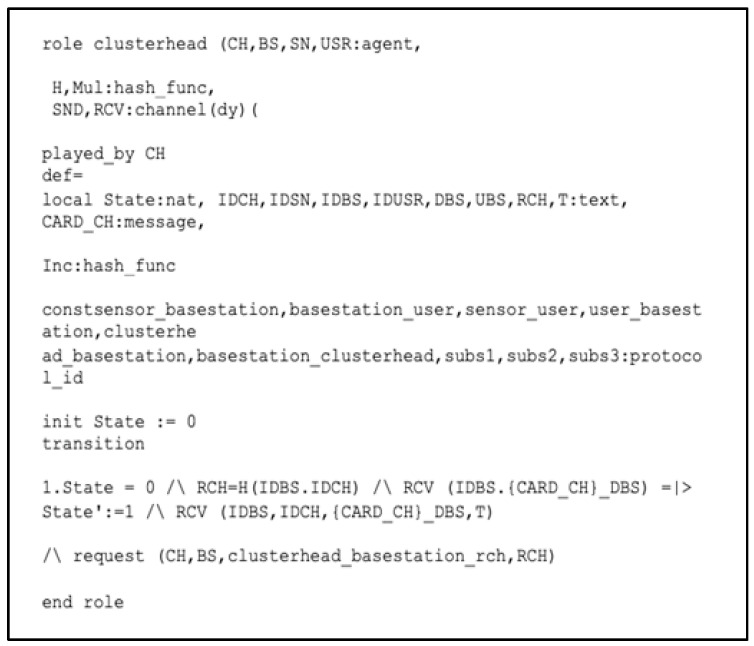
Role specification for the CH in HLPSL.

In Figure 8, we present the role of the CH, which receives a communication (ID_BS_, {cardID__USR_}_D_BS_) from the BS. After that, the CH sends a Request_of_Authentication (ID_CH_, BS_ID_, {CardID__SN_}_D_BS_, T) and request (CH, BS, clusterhead_basestaion_rch, R_CH_) messages to run the smart contract applied to the BS in order to perform the registration authentication process. The CH then receives the following information: (ID_SN_, ID_CH_, ID_BS_, {CardID__SN_}_D_BS_, T_1_) from the SN and forwards the message to the BS.

**Figure 9:**
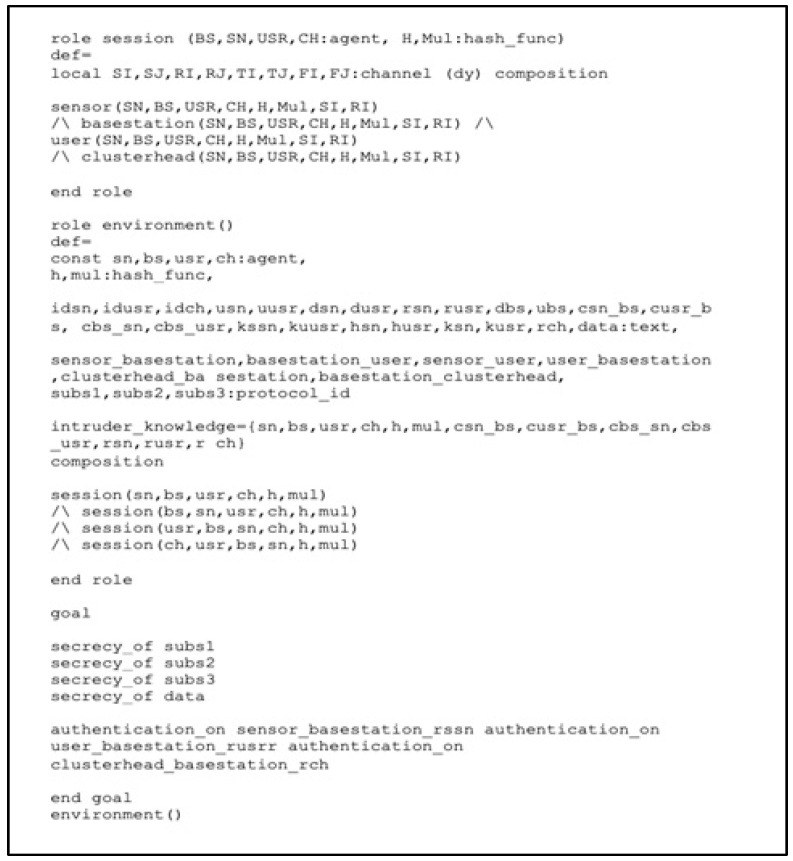
Role specification for the session, goal and environment in HLPSL.

An HLPSL language format for the roles for the session, goal, and environment is shown in Figure 9. Each role is instanced with a specific argument in the session, including the roles for the SN, CH, BS, and USR. The environment section contains a description of some fundamental constants and the composition of one or more sessions, along with some information about the intruder. HLPSL currently supports the authentication and secrecy goals that are part of the standard protocol suite. With the implementation we are performing, we are able to verify the following four secrecy goals and three authentication goals. 

#### 6.2.2. Simulation Results

**Figure 10:**
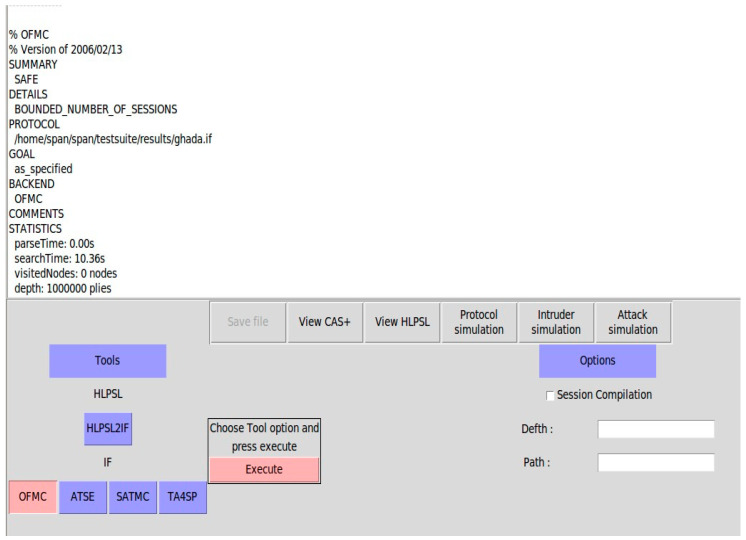
OFMC back-end simulation results.

**Figure 11:**
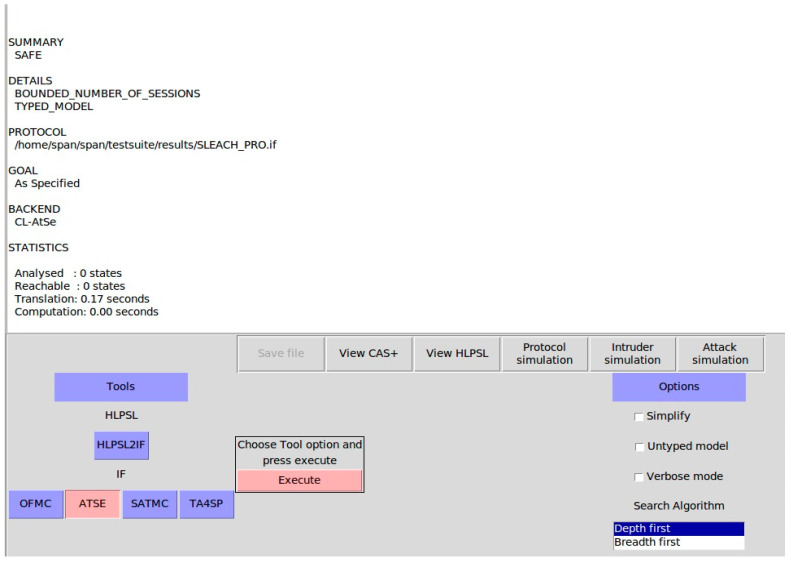
The CL-AtSe back-end simulation results.

The simulation results of the SLEACH-PRO protocol on the on-the-fly model checker (OFMC) backends and constraint-logic-based attack searcher (CL-Atse) were obtained from the AVISPA web tool. Figure 10 and Figure 11 show the security state of the SLEACH-PRO and prove that the proposed protocol is secured under OFMC and CL-AtSe, respectively. In other words, SLEACH-PRO is secure against active and passive attacks, including redo and man-in-the-middle attacks. These results correspond to the theoretical analysis and prove that the proposed SLEACH-PRO protocol is secured against attacks.

### 6.3. Informal Security Analysis

The SLEACH-PRO protocol provides a higher level of robust security protection against relevant security attacks compared to the most relevant recent schemes. Both informal and formal security analyses, such as BAN logic and the AVISPA simulation tool, are performed to evaluate the associated security of the SLEACH-PRO. Consequently, using informal analysis, we prove that the intended protocol offers protection against a variety of attacks. The authentication process of the SLEACH-PRO also is revealed using BAN logic. Moreover, the AVISPA simulation tool is used to ensure the SLEACH-PRO security features function against both replay and MITM attacks. 

#### 6.3.1. Integrity

The SLEACH-PRO ensures the message’s integrity since the transmitted message is hashed before it is transferred to the intended recipient. Both data and the hash value *h* are concatenated within the message and then are encrypted using the session key *{SN I~{(Data), (H (Data} _SN_* $\;{⇋}^{k}$*_US_}*. After receiving and decrypting the message, the recipient validates the authenticity of the received data by recalculating a new hash of the received data and then comparing it with the received *h* within the message.

#### 6.3.2. Authentication and Authorization

To authenticate entities in SLEACH-PRO, a smart contract *CardID* is generated by a BS for each node successfully registered within the network. The *CardID* includes the node’s identity, and a signed hash of the node’s identity that is signed using the BS’s private key is provided to the registered node, thus validating that the participant is initiated once a *Request_of_ Authentication (IDCH, IDBS, CardID_CH, Timestamp)* is forwarded to a BS. Any BS within the entire the network validates the smart contract determined within the received request and results in authenticating the participant if the verification process is successful.

#### 6.3.3. Confidentiality and Privacy

SLEACH-PRO ensures the confidentiality of the transferred data between the sensor node and user *{(Data), (H (Data} _SN_* $\;{⇋}^{k}$ *_US_}* using a secret key that allows for encryption and decryption processes. Thus, the SLEACH-PRO provides secure communication to prevent unauthorized access to data delivery.

#### 6.3.4. Availability

The SLEACH-PRO protocol adopts smart contracts for validating the nodes within a BS. These smart contracts effectively resist denial-of-service attacks if targeting a particular BS under certain circumstances. Another BS performs the same processes to maintain the network operation.

#### 6.3.5. Non-Repudiation

In the context of non-repudiation, users and sensors cannot argue over or change the actions they have taken or the communications they have sent. Because this protocol uses blockchain, all transactions are recorded in the blockchain as transaction records, and tampering is not permitted. The entity that later signed certain information cannot claim that it did not sign it. Similarly, a fraudulent party that only has access to the public key is unable to forge a valid signature.

#### 6.3.6. Sybil Attack 

This protocol assigns each sensor node in the network a unique identity ID_SN_, which is used (ID_SN_, ID_CH_, ID_BS_) to identify a sensor node based on its WSN subnetwork and cluster network assigned to the unique CH ID_CH,_ as well as BS ID_BS_, as well as identification of the node itself in every communication. Blockchains are used for authentication. It is thus difficult for an attacker to impersonate a genuine node in the network and communicate in the network.

#### 6.3.7. Compromised CH Attack

A compromised CH attack is a type of intrusion that tries to gain access to a CH to extract sensor node data in order to obtain sensitive information about a particular node. A SN transmits the encrypted data directly to the end user in the SLEACH-PRO protocol. Therefore, the SLEACH-PRO protocol can defend against a hacked CH attack because the data does not pass through the CH.

#### 6.3.8. Message Replay Attack

Through SLEACH-PRO’s smart contracts, authentication operations are implemented in the blockchain. Thus, a message replay attack may be possible: when a regular node requests registration from its cluster network’s CH node, it will not be re-registered at this time since the timestamp in the authentication message indicates that the node has completed registration. Due to this, message replay cannot be used by an attacker for authentication. Additionally, digital signatures and hashing functions are employed to detect data modification by the attacker.

#### 6.3.9. Key-Compromise Impersonation Attack

If Î knows SN’s private key, he can impersonate the USR to the SN, according to the key-compromise impersonation attack. The SLEACH-PRO protocol, on the other hand, prevents Î from doing this. Assume the SN’s private key dSN has been hacked, and Î desires to impersonate the SN. Î must have a valid K_USR_ = d_USR_.U_BS_. He will not be able to authenticate himself to BS otherwise. It is plausible if he has access to the USR’s/BS’s private key, but Î was unable to deduce d_USR_ = d_BS_ from U_USR_ = U_BS_ owing to SLEACH- PRO infeasibility. As a result, the proposed protocol guards against key compromise impersonation.

#### 6.3.10. Denial of Service

SLEACH-PRO is a protocol that enables people, for example hackers, to submit a transaction authentication request per transaction, but it needs some resources to do so. Accordingly, attackers will not be able to overload the blockchain by submitting a lot of authentication requests. This is because, in the SLEACH-PRO protocol, the blockchain is applied to more than one BS. Therefore, if an attacker performs a DOS attack on a particular BS, another BS can perform the same function. Thus, the SLEACH-PRO protocol can resist a DOS attack.

#### 6.3.11. Man in the Middle Attack 

Suppose the attacker intercepts the authentication message sent during authentication and uses a third party to conduct the attack. The third party can never hack the authentication process, just as if the attacker were parsing the message resend attack. In this way, the SLEACH-PRO protocol is capable of withstanding man-in-the-middle attacks.

#### 6.3.12. Spoofing Attack

SLEACH-PRO’s authentication mechanism requires that every connection verify the CardID to prove the connection’s unique identity, since every connection must authenticate itself via identity authentication. A node cannot be attacked by concealing its identity, thus making it impossible for an attacker to launch attacks. This means that SLEACH-PRO is impervious to spoofing attacks.

#### 6.3.13. Message Replacement Attack 

An attacker cannot disguise another node’s identification in order to attack it with its unique identity. Since the authentication method proposed in this research entails sending the CH node registration to the blockchain right away, during communication, the chances of attack by message substitution are eliminated. During the registration phase, the smart contract verifies the node’s registration. Although the message can be altered, the metadata contains the original node’s identifying information. The CH can reject authentication requests, and communications sent from normal nodes that join the network can be blocked.

## 7. Analysis of Performance

In this part, the performance related to the computational cost, communication costs, and security properties of the SLEACH-PRO are examined and compared with the most relevant recent schemes [22,23,24,25,26,27,28,29,30,31,32]. Table 5 lists the computational cost notations and the execution time for each cryptographic operation [33].

Table 6 shows the computational cost required for the registration and authentication phases of SLEACH-PRO protocol and its compared to other related protocols. Ref. [33] indicates that *T*_h_ takes 0.0023 ms to perform, *Te* takes 0.0046 ms to perform, *T*_PM_ takes 2.226 ms and *T*PA takes 0.0288 ms to perform. As a result, we calculate that the SLEACH-PRO protocol takes 0.0506 ms to execute. We compared the execution time of our protocol with the execution time of other protocols in Table 6.

As seen in Table 6, the SLEACH-PRO protocol has the lowest computational cost compared to [22,23,24,26,27,28,29,31,32]. In [22], two keys are pre-loaded on each sensor node (SN): the master key is used exclusively by the CH to communicate with it, and the BS key is used to communicate with SN. This method is called one-way key hashing. The session key generation stage is the only stage using public key encryption. The remaining stages use symmetric encryption/decryption. In [23], a lightweight authentication mechanism is relied upon, and ECC is relied upon with pre-shared keys. Here, nodes that do not participate in the transmission of data are not authenticated with all their neighbors, whereas in [24] only elliptic curve coding (ECC) is relied upon. In [26], response is based on smart card-based symmetric key encryption in addition to using the hash function. Moreover, in [27], two-way authentication and the hashing function are issued in addition to the smart card, whereas in [28], cipher is used with the elliptical curve function, symmetric key, mac function, and hash function. Another approach [29] uses only one-way hashing and xor operations, while [31] relies on xor operations, one-way hash functions, and lightweight authentication. One-way hash functions are less expensive than ECC or encryption/decryption processes. [32] relies on the elliptic curve function, the hash function, and the symmetric encryption/decryption.

SLEACH-PRO has the highest computational cost compared to [25,30]. In [25], the authors used keyed hashing (HMAC) functions with basic symmetric encryption/decryption and a two-way encryption algorithm to minimize the cryptographic burden as it is performed once per authentication request, whereas in [30], only one-way hash and xor operations are used. However, the general increase in the proposed protocol is justified because the proposed SLEACH-PRO protocol relies on blockchain technology and additional processes, such as the ECC algorithm and hashing functions, to increase security and provide better security features as well as resistance to many attacks, as shown in Table 7.

## 8. Conclusions and Future Work

Since the LEACH-PRO protocol has several benefits, including energy efficiency, scalability, low complexity, and durability for WSNs, this paper introduces a new variant of the LEACH-PRO protocol by adopting the blockchain security technique to protect the WSNs. The proposed SLEACH-PRO protocol performs a decentralized authentication mechanism by applying a blockchain to multiple base stations to avoid system and performance degradation in the event of a station failure. In addition, the formal security analyses of the SLEACH-PRO, using both the AVISPA tool and BAN logic, are proven to ensure that the SLEACH-PRO is safe against passive and active attacks. As a result, the SLEACH-PRO achieves a better security level and efficient communication services compared to existing related WSNs’ security protocols, making the SLEACH-PRO able to be used efficiently in several applications in future smart cities. In future work, network simulation based on the SLEACH-PRO protocol will be considered to measure the network performance and design a novel scheme that is suitable for utilization in WSNs.

## Figures and Tables

**Table 1:** Secure routing protocol using blockchain in WSN.

Reference	Network	Year	Security Algorithm	Result
[12]	Wireless Sensor Network	2021	Attribute-based blockchain Encryption.	Higher performance compared to other methods mentioned in their research.
[13]		2021	Based on CAN for authenticating WSN nodes, SHA256 for verifying messages and cooperating CH responsible for communication with BS. Blockchain with Encryption	Performance improvement for secure routing and increased data security performance.
[14]		2019	For routing nodes to identify the next nod, reinforcement learning method was used. Records were kept of routing information contracts that are intelligent. Blockchain essentially functions as a database. Use of distributed ledgers. This algorithm is more efficient at processing transactions.	Their system protects against malicious node attacks, throughput and latency are excellent.
[15]		2021	The power and reliability of routing information was increased by combining Markov with blockchain, which is a distributed ledger with decentralization, Tamper resistance, and traceability features.	Hostile node attacks were eliminated with ease, and device latency was excellent.
[16]		2021	A decentralized authentication mechanism, based on a blockchain.	The BSI protocol is efficient and robust, which speeds up computing time and reduces power consumption
[18]		2020	Hybrid blockchain-based authentication and trust evaluation mechanism. The private blockchain is applied to CH while the public blockchain is applied to BS; the smart contract makes sure whether the CH node exists or not.	High throughput, packet delivery and can deal with malicious nodes effectively.

**Table 2:** Notations and respective descriptions.

Notations	Descriptions
*SN*	Sensor node
*USR*	End user
*BS*	Base station
*CH*	Cluster head
*Q*	A large prime number of k-bit length and *q* > 3
*F* *q*	A finite field
*Eq (a, b)*	An elliptic curve defined over on *Fq* with prime order *n*
*K*	The security parameter
*C*	The certificate based on ECC
*R_SN_*	Random point of the sensor node
*R_USR_*	Random point of the user
*R_CH_*	Random point of the cluster head
*Q*	A base point of order n over Eq(a, b)
*(d, U)*	The private/public key pair of the entity, where U = d .Q
*H()*	One-way cryptographic hash function
*ID_SN_*	Identity of sensor node
*ID_USR_*	Identity of end user
*ID_BS_*	Identity of base station
*ID_CH_*	Identity of cluster head
*SHK*	Shared key between the sensor node and user
*Î*	The Adversary

**Table 3:** Notations used in BAN logic.

Symbol	Usage
SN, CH, BS, USR	Principle
$\overset{K}{\to }N$	Public key
${⇋}^{k}$ _N_	Shared key
$K_{N}^{-1}$	Private key
{M}_KN_	Message encrypted by public key
ECC-P	Elliptic curve parameter
T_N_	Timestamp

**Table 4:** Assumptions used in SLEACH-PRO.

Symbol	Usage
BS |≡ |$\overset{K}{\to }$SN	The public key of node SN believes the BS
BS |≡ |$\overset{K}{\to }$USR	The public key of node USR believes BS
SN |≡ BS |~ |$\overset{K}{\to }$SN	If just the nodes are signed and have been provided the BS’s public key, the BS believes node SN.
C |≡ BS |~ |$\overset{K}{\to }$CH	If just the nodes are signed and have been provided the BS’s public key, the BS believes node CH.
C |≡ BS |~ |$\overset{K}{\to }$USR	If just the nodes have been signed and given the BS’s public key, the BS believes node USR.

**Table 5:** Execution time of different cryptographic operations (ms).

Notation	Description	Execution Time (ms)
*T_h_*	Execution time of the hash function	0.0023
*T*_*e*_	Execution time of the symmetric encryption/decryption	0046
*T*_*P*M_	Execution time of the elliptic curve point multiplication	2.226
*T_PA_*	Execution time of the elliptic curve point multiplication	0.0288

**Table 6:** Computational cost comparison.

Protocol	Total Computation Time	Total Cost
Chatterjee et al. [22]	4T_h_ + 3T_PM_ + 3T_PA_ + 7T_e_	6.8058 ms
Razali et al. [23]	6 T_h_ + 12 T_e_	0.069 ms
Gupta [24]	5T_h_ + 3T_PM_ + 3T_PA_ + 5T_e_	6.7989 ms
Qin et al. [25]	4T_h_ + 6T_e_	0.0368 ms
Lu et al. [26]	27T_h_ + 12T_e_ +	0.1173 ms
Farash et al. [27]	36T_h_ + 4T_e_	0.1012 ms
Porambage et al. [28]	6 T_h_ + 4T_PM_ + 2T_PA_	8.9754 ms
Kumari and Om [29]	24 T_h_	0.0552 ms
Vaidya et al. [30]	11 T_h_	0.0253 ms
Dhillon and Kalra [31]	23 T_h_ + 4T_e_	0.0759 ms
Rangwani et al. [32]	15T_h_ + 4T_PM_ + 4T_e_	8.9560 ms
SLEACH-PRO	22 T_h_ + 1 T_e_ + 10 T_PM_	0.0506 ms

**Table 7:** The evaluation of the SLEACH protocol.

Protocols	SF_1_	SF_2_	SF_3_	SF_4_	SF_5_	SF_6_	SF_7_	SF_8_	SF_9_	SF_10_	SF_11_	SF_12_	SF_13_	SF_14_	SF_15_
Cui et al. [18]	✓	✓	✓	✓	✓	✓	✓	✓	-	-	✓	✓	✓	☓	☓
Chatterjee et al. [22]	☓	-	☓	☓	✓	☓	☓	✓	☓	☓	-	-	✓	✓	☓
Gupta [24]	✓	-	✓	✓	✓	☓	☓	☓	✓	-	✓	✓	✓	☓	✓
Qin et al. [25]	-	-	✓	✓	✓	☓	☓	☓	✓	☓	✓	✓	✓	☓	☓
Lu et al. [26]	-	-	-	-	☓	✓	✓	☓	✓	-	-	✓	✓	✓	✓
Farash et al. [27]	-	-	-	✓	✓	✓	✓	✓	-	-	✓	✓	✓	✓	✓
Porambage et al. [28]	-	-	-	☓	☓	✓	✓	✓	✓	✓	☓	✓	✓	☓	☓
Kumari and Om [29]	✓	-	-	✓	-	-	✓	☓	✓	-	✓	✓	✓	✓	✓
Vaidya et al. [30]	✓	-	-	✓	✓	-	✓	✓	✓	✓	☓	☓	✓	☓	☓
Dhillon and Kalra [31]	-	-	-	✓	✓	✓	✓	☓	-	✓	✓	✓	✓	☓	✓
Rangwani et al. [32]	-	-	-	✓	✓	✓	✓	✓	-	✓	✓	✓	✓	☓	✓
SLEACH-PRO	✓	✓	✓	✓	✓	✓	✓	✓	✓	✓	✓	✓	✓	✓	✓

SF_1_—Compromised CH Attack, SF_2_—Non-repudiation Attack, SF_3_—Sybil Attack, SF_4_—Message Replay Attack, SF_5_—Man in the Middle Attack, SF_6_—Denial of Service Attack, SF_7_—Spoofing Attack, SF_8_—Key-Compromise Impersonation Attack, SF_9_—Message Replacement Attack, SF_10_—Parallel session attack, SF_11_—User anonymity, SF_12_—Sensor anonymity, SF_13_—BAN logic is used to perform security analysis, SF_14_—Authentication mechanism, SF_15_—The AVISPA tool is used to perform formal security verification, - = Not applicable in the protocol, ✓ = Secure against attack, ☓ = Insecure against attack.

## Data Availability

Not applicable.
